# Sequence Effect on the Activity of DNAzyme with Covalently Attached Hemin and Their Potential Bioanalytical Application

**DOI:** 10.3390/s22020500

**Published:** 2022-01-10

**Authors:** Joanna Kosman, Krzysztof Żukowski, Andrea Csáki, Wolfgang Fritzsche, Bernard Juskowiak

**Affiliations:** 1 Department of Bioanalytical Chemistry, Faculty of Chemistry, Adam Mickiewicz University, Uniwersytetu Poznańskiego 8, 61-614 Poznan, Poland; krzysztof.zukowski@amu.edu.pl (K.Ż.); juskowia@amu.edu.pl (B.J.); 2 Leibniz Institute of Photonic Technology, Albert-Einstein-Strasse 9, 07745 Jena, Germany; andrea.csaki@leibniz-ipht.de (A.C.); wolfgang.fritzsche@leibniz-ipht.de (W.F.)

**Keywords:** covalent DNAzyme, G-quadruplex, hemin, DNA detection

## Abstract

In this work we investigated the effect of a DNA oligonucleotide sequence on the activity of a DNAzyme with covalently attached hemin. For this purpose, we synthesized seven DNA-hemin conjugates. All DNA-hemin conjugates as well as DNA/hemin complexes were characterized using circular dichroism, determination of melting temperatures and pKa of hemin. We observed that hemin conjugation in most cases led to the formation of parallel G-quadruplexes in the presence of potassium and increased thermal stability of all studied systems. Although the activity of DNA-hemin conjugates depended on the sequence used, the highest activity was observed for the DNA-hemin conjugate based on a human telomeric sequence. We used this DNAzyme for development of “sandwich” assay for detection of DNA sequence. For this assay, we used electric chip which could conduct electricity after silver deposition catalyzed by DNAzyme. This method was proved to be selective towards DNA oligonucleotides with mismatches and could be used for the detection of the target. To prove the versatility of our DNAzyme probe we also performed experiments with streptavidin-coated microplates. Our research proved that DNAzyme with covalently attached hemin can be used successfully in the development of heterogeneous assays.

## 1. Introduction

Bioanalytical approaches rely on the use of sophisticated equipment (e.g., qPCR, LC-MS, SPR), specific reactions, or molecular probes. Recently, the focus of researchers concentrated on the development of bioanalytical methods that do not require sophisticated equipment and are easy to transfer to point-of-care diagnostics. For this purpose, probes are designed that connect recognition of analyte and generation of analytical signal (colorimetric or fluorescence). Development of the new bioanalytical methods is often achieved using DNAzymes [1,2]. These catalytically active DNA molecules can catalyze such reactions such as DNA and RNA ligation or cleavage, DNA phosphorylation, porphyrin metalation, or peroxidase reaction [3,4,5]. In many bioassays G-quadruplex/hemin DNAzyme with peroxidase activity is used for the analytical signal generation [6]. Such a DNAzyme is formed by G-quadruplex (G4) and hemin molecule, which interact by end-stacking mode [7]. DNA G-quadruplexes are DNA tertiary structures formed by DNA oligonucleotides rich in guanosine residues [8]. Four guanosine residues form G-tetrad stabilized by Hoogsteen hydrogen bonds. Additionally, the G4 structure is stabilized by stacking interactions between G-tetrads and coordination of cations, mainly K^+^ or Na^+^. External G-tetrads are able to interact with planar molecules, such as hemin, through end-stacking interactions. DNAzymes possess many advantages over protein enzymes such as simple synthesis and purification, thermal stability and reversibility, simple modification and possibility to design complicated constructs thanks to hybridization between complementary strands. G-quadruplex/hemin DNAzyme mimics horseradish peroxidase (HRP) and catalyzes reaction between hydrogen peroxide and organic substrate. By choosing the appropriate substrates, DNAzymes can generate colorimetric, fluorescence, chemiluminescence or electric signal [6,9]. 

Despite many advantages of the association-type G-quadruplex/hemin DNAzymes, there is still a need for improvement of basic functions of these systems. One of the issues is their lower catalytic activity comparing with HRP; another problem is the necessity of considering a blank value of hemin. In the case of association DNAzyme system, hemin must be added to the negative control and can generate a blank signal (since hemin itself possesses residual peroxidase activity). To eliminate the problem of blank value and to improve the activity of DNAzyme, a strategy was advised to attach covalently hemin to G-quadruplex DNA. Two approaches were developed for hemin conjugation to DNA: amino-coupling or “click” reaction. The first attempts of hemin conjugation to DNA oligonucleotide were carried out by the Niemeyer group [10]. However, their main goal was to reconstruct enzymes with protein molecules [10,11,12]. Later on, Thirstrup and Baird were synthesized DNAzymes with covalently attached hemin and it was proved that conjugates maintained activity even at conditions where the formation of G4 was not favored [13]. On the other hand, Nakayama et al. used DNA-hemin conjugates to get insight into the exact role of topology and mechanism of DNAzyme action [14]. DNAzymes with covalently attached hemin were also demonstrated to possess higher electrocatalytic activity than G4/hemin complexes [15]. All of these studies were performed using DNA-hemin conjugates obtained by amino-coupling reaction. In our previous research we have developed alternative method for hemin conjugation to DNA using click chemistry. We have also compared both methods of DNA-hemin conjugation [16,17]. However, only hemin aptamers (PS2.M and CatG4) were studied after conjugation with hemin molecule. DNAzymes based on those oligonucleotides have relatively high activity even before hemin conjugation. Here, we decided to expand our studies using DNAzymes based on DNA oligonucleotides that form association DNAzymes possessing low activity. For this purpose, we decided to use DNA oligonucleotide based on the human telomeric sequence, thrombin-binding aptamer (TBA), and a reference oligonucleotide which is not able to form G-quadruplex. This is important since previously studied DNAzymes were based on similar DNA oligonucleotides and sequence diversity is an important factor in the development of DNAzymes for application in bioanalysis. We have also taken first steps in the development of heterogeneous bioassay using DNAzymes with covalently attached hemin. Application of DNA-hemin conjugates in bioassays development is beneficial since conjugates usually possess higher activity, no hemin is added to blank probe what reduces blank signal. Finally, covalent conjugates are more suited for heterogeneous applications in which multiple washing steps can cause hemin wash out in the case of complex G-quadruplex/hemin approach. 

## 2. Materials and Methods

### 2.1. Materials

DNA oligonucleotides (HPLC purified) used in this study (Table 1) were purchased from Genomed S.A. (Warsaw, Poland) or Metabion (Planegg, Germany) and were used without further purification. The concentration of DNA was quantified by UV-Vis spectroscopy at 85 °C with the following extinction coefficients at 260 nm (M^−1^, cm^−1^) A = 15,400, T = 8700, G = 11,500, C = 7400 [18]. Amplex red, 2,2′-azinobis(3-ethylbenzothiozoline)-6-sulfonic acid (ABTS), 4-(1-metylohydrazyno)-7-nitrobenzofurazan (MNBDH), hemin and H_2_O_2_, were purchased from Sigma-Aldrich (Poznań, Poland). Hemin was dissolved in DMSO and the 1 × 10^−2^ M stock solution was stable for at least one month. The Silver Enhancing Kit for Optical and Electron Microscopy was purchased from BBI Solutions (Cardiff, UK). Other reagents were of analytical grade and used as received. DNA-hemin conjugates were synthesized according to our previously reported procedure [16], purified using HPLC and characterized using MALDI-TOF mass spectrometry. High-capacity plates coated with streptavidin (96-well) were purchased from Thermo Fisher Scientific (Waltham, MA, USA).

### 2.2. Circular Dichrosim (CD) Spectra 

CD spectra were measured on a J-1500 spectropolarimeter (Jasco, Tokyo, Japan) with 100 nm/min scanning speed and bandwidth of 1 nm. Spectra were recorded in quartz cells of 1 cm path length and averaged from three scans. All samples contained 2 µM G4 oligonucleotide, 10 mM Tris-HCl buffer pH = 8.0, and required cation at adequate concentration. Prior to the experiments, the samples were heated at 95 °C for 5 min and then cooled on ice for 15 min.

### 2.3. Melting Profiles

The melting profiles of G-quadruplexes followed by CD spectrometry were measured by heating/cooling of samples with monitoring ellipticity at 265 nm (parallel G4) or 295 nm (antiparallel or hybrid G4). All experiments were performed in quartz cells of 1 cm path length. Before measurements, oligonucleotide samples were denatured at 90 °C for 5 min. All samples used in this study contained 2 μM G4 DNA, 10 mM Tris-HCl buffer pH = 8.0 and required salt concentration.

### 2.4. Activity Measurment

The samples containing 1 µM G4 oligonucleotide or G4-hemin conjugate, 10 mM Tris-HCl buffer pH = 8.0, 1 µM hemin (if needed) and salt at adequate concentration, were heated at 95 °C for 5 min. Next, samples were incubated for 15 min on ice bath and allowed to obtain room temperature. After addition of indicator (ABTS, MNBDH, or Amplex Red (in the case of fluorogenic substrates 50 nM DNA and hemin were used)), samples were transferred on a microplate and addition of H_2_O_2_ initiated the peroxidation reaction. The changes of absorbance (ABTS, λ = 417 nm) or fluorescence (MNBDH, λ_ex_ = 470 nm, λ_em_ = 560 nm; Amplex Red, λ_ex_ =560 nm, λ_em_ = 590 nm) were monitored, over reaction time (10 min) using microplate reader Tecan M2000 (Austria). 

### 2.5. DNA Detection Procedure with Electric Chip

Chips were prepared according to procedure described by Moller et al. [23]. Briefly, chips were silanized using 10 mM GOPS ((3-glycidyloxypropyl)trimethoxysilane) in toluene by incubation overnight in RT and then heating for 3 h in 70 °C. After the silanization chips were washed with toluene, ethanol, and water and dried under nitrogen. Next, 1 µM amino-modified oligonucleotide (SC) in 0.1 M KOH was immobilized on the surface of the chip by manual spotting between electrodes. Chip was then incubated overnight in RT followed by backing in 80 °C. Chip was then washed with MQ water and dried under Ar. Next, the surface of the chip was blocked using 50 mM solution of ethanolamine with 0.1% SDS in 0.1 M Tris-HCl pH = 9.0. After washing with Tris-HCl buffer and drying, the DNA analyte (HIV) in various concentrations (10^−6^–10^−14^ M) was manually spotted on the chip. Hybridization was carried out for 2 h in 36 °C in humidity chamber. After hybridization, chip was washed with 2× and 0.5× SSC buffer and dried under N_2_ before the next step of hybridization with S2 probe. After both hybridization steps, silver deposition was performed using silver enhancing kit (BBI solutions) and incubation with the solution for 10 min. Finally, chip was washed with water, dried under N_2_ and the resistance was measured using chip reader.

### 2.6. DNA Detection Procedure with Microplate Approach

Selected wells of 96-well plate (streptavidin coated) were washed three times with buffer (10 mM Tris-HCl, 100 mM KCl, pH = 7.2). Then, 100 µL of 5 µM biotin-SC was added to the wells and plate was incubate for 2 h in RT with shaking. Wells were then washed with buffer three times. Next, 50 nM DNA analyte (HIV) was added to the selected wells (positive control) and microplate was incubated for 2 h in 36 °C with shaking. Wells were then washed with buffer three times. Next, 1 µM solution of S2 probe was added to the wells, followed by incubation for 2 h in 36 °C (with shaking) and washing of wells three times with buffer. In the final step, 1 µM solution of Amplex Red in buffer was added to the wells and the peroxidase reaction was started by addition of H_2_O_2_ (1 mM) to the wells followed by measurement of fluorescence (λ_ex_ = 560 nm, λ_em_ = 590 nm).

## 3. Results

### 3.1. Effect of the DNA Sequence on the DNAzyme Properties

**Figure 1:**
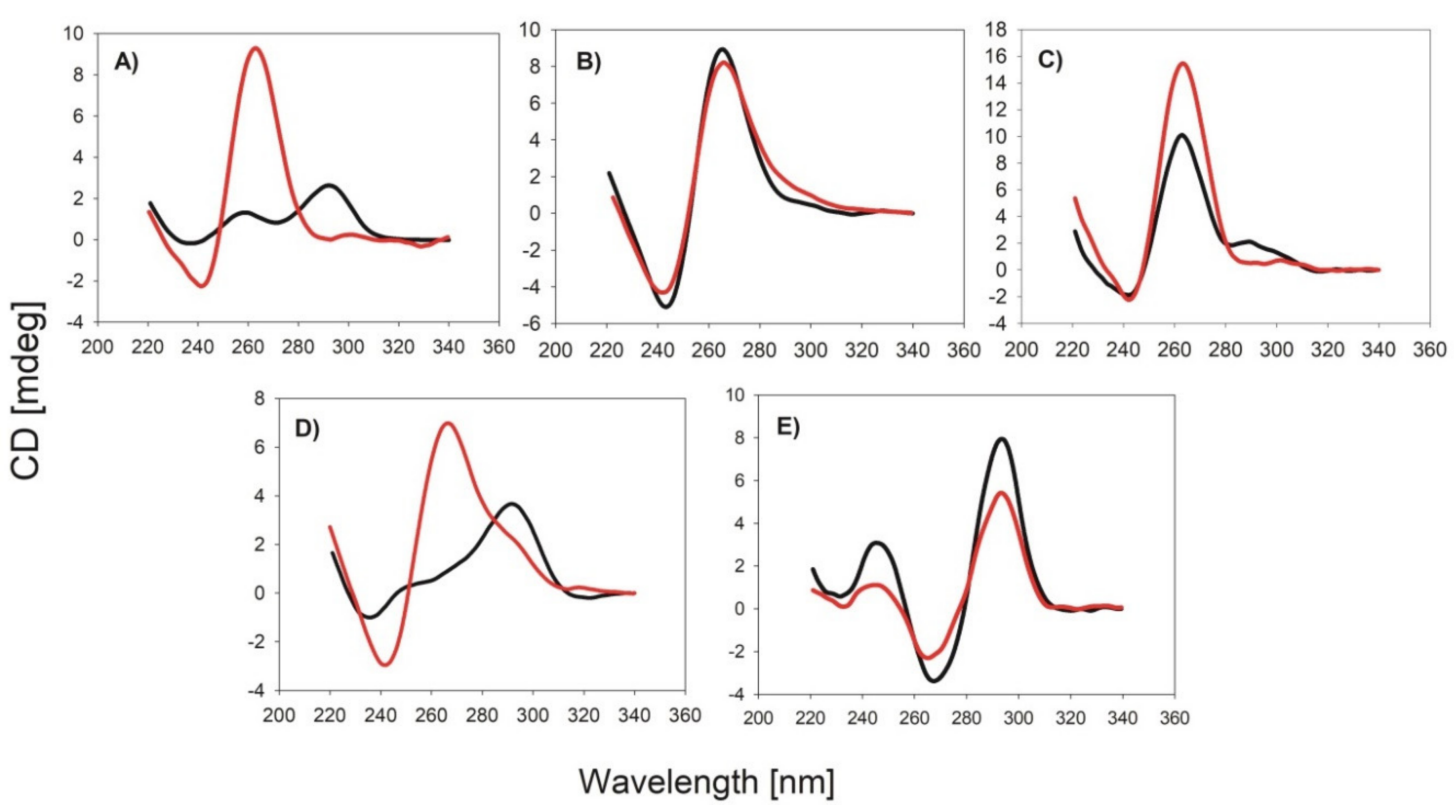
CD spectra of G4-hem conjugates (red) and G4 oligonucleotides (black) for PS2.M (**A**), CatG4 (**B**), AGRO100 (**C**), HT22 (**D**) and TBA (**E**). Conditions: 2 µM DNA, 100 mM KCl, 10 mM Tris-HCl (pH = 8.0).

The first part of our study was focused on investigation of DNA oligonucleotide sequence effect on the topology of G4, melting temperature, pKa, and peroxidase activity of DNA-hemin conjugates. We performed experiments using six oligonucleotides: PS2.M and CatG4, which are known aptamers for hemin and together with AGRO100 are typically used for obtaining DNAzymes [7,19,24], HT22 possessing sequence of human telomeric DNA and containing three TTAGGG repeats, TBA that is an aptamer for thrombin, and (Ref) as a reference oligonucleotide that is unable to form G-quadruplex structure. Sequences of studied oligonucleotides are shown in the Experimental section in Table 1. In order to determine the topology of studied oligonucleotides and DNA-hemin conjugates we used circular dichroism technique (CD). This technique allows to distinguish between basic G-quadruplex topologies: parallel (positive band at 265 nm and negative band at 240 nm), antiparallel (positive band at 295 nm an d negative band at 265 nm), and hybrid (positive band at 295 nm with a shoulder at 265 nm and negative band at 240 nm). PS2.M is known to form hybrid structure or a mixture of several topologies [25,26]. As it can be seen in Figure 1A, covalent attachment of hemin to PS2.M caused transformation of DNA topology to the parallel G-quadruplex. In the case of CatG4 and AGRO100 sequences the topology of G-quadruplexes were already parallel and conjugation of hemin did not affect their G-quadruplex topology (Figure 1B,C). HT22 forms hybrid topology in the presence of potassium cations and interestingly, covalent attachment of hemin forced formation of parallel G-quadruplex (Figure 1D). TBA, unlike the others studied oligonucleotides, forms G-quadruplex with only two tetrads (other studied G4s have three tetrads) and this is the only oligonucleotide which, after hemin conjugation, did not form parallel G-quadruplex and maintained antiparallel topology (Figure 1E). We have also measured CD spectra of studied oligonucleotides and conjugates in the presence of sodium cation (Figure S1). In the case of PS2.M and CatG4 in NaCl, we have observed shift to parallel topology after hemin conjugation. AGRO100 formed already parallel G4 and hemin conjugation led to the increase of CD signal intensity but this did not caused shift of topology. In contrast, TBA and HT22 in NaCl formed mainly antiparallel G-quadruplexes even after hemin conjugation. 

In order to further characterize DNA-hemin conjugates and their oligonucleotides counterparts, we determined melting temperatures for oligonucleotides alone, oligonucleotides/hemin complexes, and oligonucleotide-hemin conjugates (Table 2). Melting temperatures were determined using the CD technique, and for parallel topologies melting profiles were monitored at 265 nm and for antiparallel and hybrid topologies, at 295 nm. We have observed that attachment of hemin caused an increase of the thermal stability of all conjugates in the presence of potassium cation and the T_m_ (melting temperature) were above 80 °C. We were not able to determine exact values of T_m_ since incomplete melting profiles were registered in the measured temperature range (10–90 °C). For association complexes of G4 with hemin in the presence of potassium it can be seen that binding of hemin also caused increase of T_m_ values from 0.7 to 8.1 °C. These increases, however, were not so spectacular as those for DNA-hemin conjugates, for which T_m_ increased by at least 15 to 30 °C. In the presence of sodium cation, association of hemin did not affect melting temperatures. In contrast, in the case of DNA-hemin conjugates, increase of thermal stability was observed. This is especially visible for TBA oligonucleotide, which in the sodium does not form G-quadruplex since its T_m_ is below room temperature. However, conjugation of hemin to TBA resulted in the remarkable increase of T_m_ by 25.7 °C. These results provide clear evidence that conjugation of hemin can improve the thermal stability of G-quadruplex structures for all studied sequences.

Many recent studies on peroxidase-mimicking DNAzymes focus on identifying the factors that are responsible for different activity of DNAzymes formed by particular sequences [19,27]. One of the main factors influencing DNAzyme activity is G-quadruplex topology, since binding of hemin is preferential for topologies with exposed G-tetrad and external loops (parallel topology). However, this relation is not always straightforward and in some cases parallel G-quadruplexes do not have the highest activity; sometimes hybrid or mixed topologies can have higher activity than DNAzymes based on parallel G-quadruplexes [25]. The other factor related to high catalytic activity of DNAzymes is pKa of hemin. It was proved that the high pKa of hemin is connected with higher peroxidase activity of DNAzymes [28]. We measured pKa of hemin for G4/hemin complexes and G4-hemin conjugates. As it can be seen in Table 3, the highest values of pKa for G4/hemin complexes were observed for PS2.M and CatG4 based systems. These sequences were tailored as hemin aptamers and are known to form DNAzyme with high activity. AGRO100 also exhibited relatively high pKa. In contrast, pKa values determined for complexes of hemin with HT22 and TBA are lower. For conjugates of PS2.M-hemin and CatG4-hemin we observed decrease of pKa values. For AGRO100-hemin the values did not change significantly. Interestingly, the substantial increase of pKa value was observed for conjugates of hemin with HT22 and TBA. 

**Figure 2:**
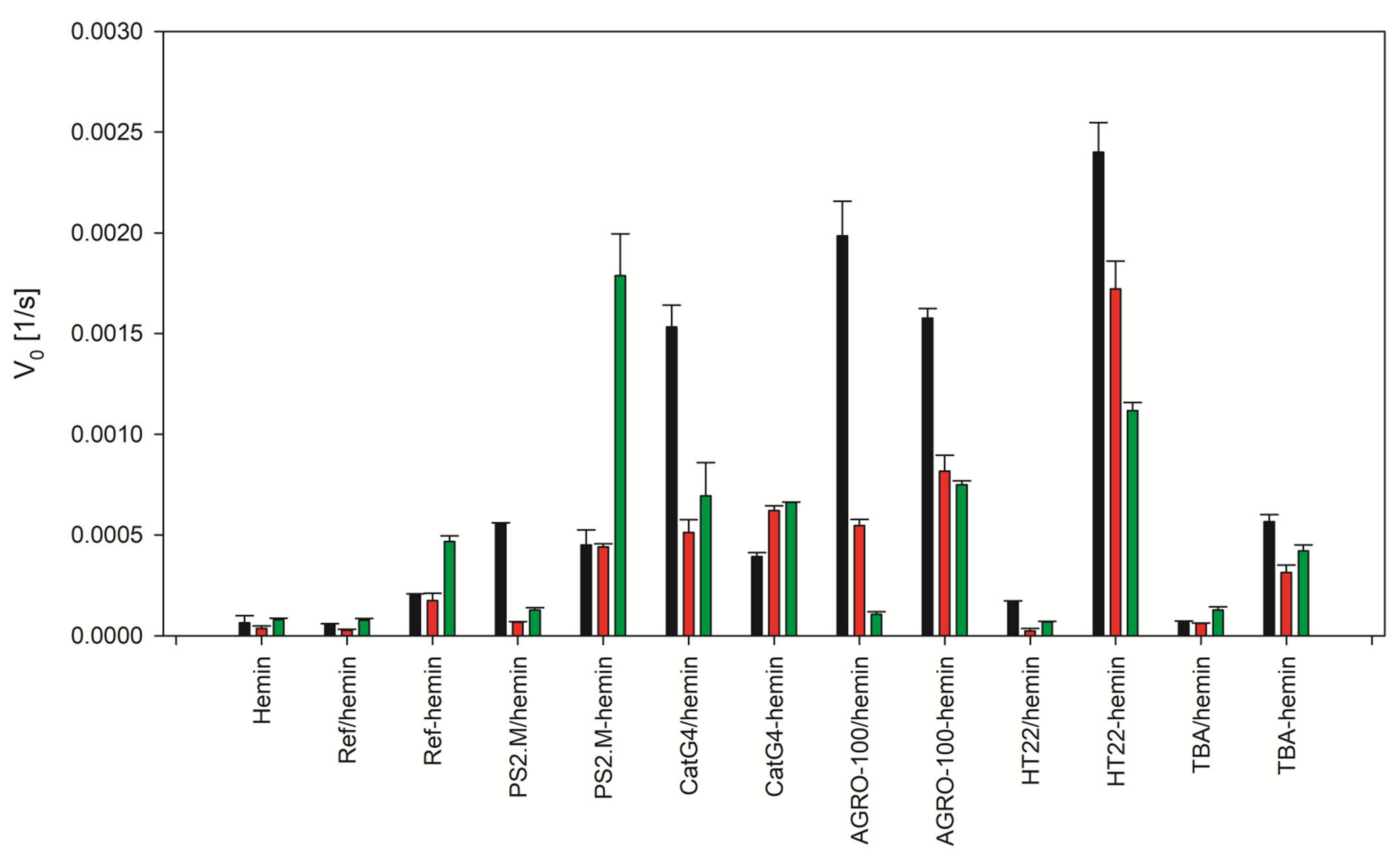
Initial velocity of ABTS per oxidation by hemin, DNA/hemin complexes and DNA-hemin conjugates in various cationic conditions: 100 mM KCl (black), 100 mM NaCl (red), without cations (green).

The final step of the study devoted to the sequence effect on the performance of DNA-hemin conjugates was measurement of their peroxidase activity in comparison to DNA/hemin complexes (Figure 2). As expected, reference sequence Ref did not form DNAzyme and possessed residual catalytic activity comparable to that for hemin alone. PS2.M, CatG4 and AGRO100 formed association complexes with hemin that possessed high activity in the presence of potassium and lower activity in the presence of sodium or without stabilizing cations. HT22 and TBA formed G4/hemin complexes with very low peroxidase activity. In the case of DNA-hemin conjugates, we have observed unexpected increase of peroxidase activity after conjugation of hemin to the reference oligonucleotide. This issue we have further investigated and it will be addressed in the next section of the manuscript. The PS2.M-hemin exhibited lower activity in the presence of potassium than PS2.M/hemin but some increase in activity was observed for samples in the presence of sodium and very high increase of activity was observed for conditions without stabilizing cations. For CatG4-hemin, the activity decreased significantly in the presence of potassium but minimal differences were observed for other cationic conditions. The activity of Agro100-hemin in KCl solution was slightly lower than that for Agro100/hemin system but increase was observed for the samples in the presence of sodium and without any cation. The highest increase of activity was observed for conjugates of hemin with HT22 and TBA. For HT22-hemin the activity in the presence of potassium was 13.5 times higher compared to that for HT22/hemin and for TBA-hemin the activity increased by 7.5 times. 

**Figure 3:**
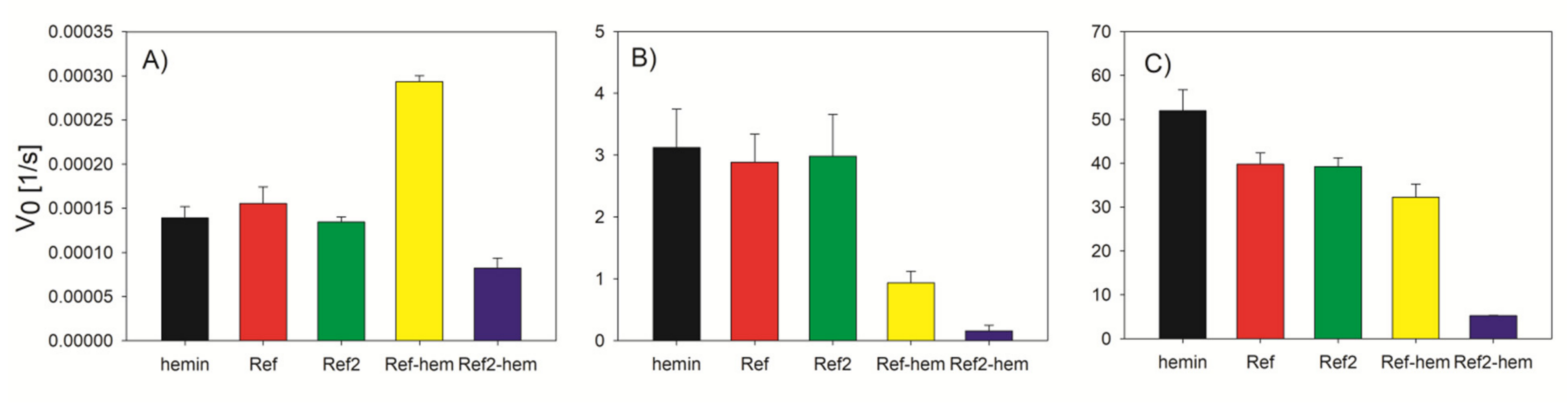
Initial velocity of substrates oxidation by reference oligonucleotides Ref and Ref2 in complex with hemin and DNA-hemin conjugates in comparison to hemin: (**A**) ABTS, (**B**) MNBDH, and (**C**) Amplex Red.

Enhanced activity observed for the conjugate of reference oligonucleotide that is not able to form G-quadruplex structure encouraged us to investigated this issue further. Since the sequence was designed as a random coil using the mixture of all nucleotides, we suspected that guanine residues present in Ref. could take part in the catalytic process. To verify this hypothesis, we decided to design additional reference sequence containing only adenosine and thymidine residues (Ref2). We have investigated the activity of Ref and Ref2 complexes with hemin and the conjugates using three different substrates: ABTS, Amplex Red and MNBDH (Figure 3). For the reaction of ABTS peroxidation, we observed an increase of catalytic activity for Ref-hem but not for the Ref2-hem. The activity of Ref2-hem with ABTS was even lower than that for hemin alone. In the case of the associate complexes with hemin, the activity for both reference oligonucleotides was comparable to that for hemin. One can conclude that hemin does not form complexes with single-stranded reference oligonucleotides tested and activity of these samples comes from free hemin. In the case of fluorogenic substrates the activity of hemin/reference sequence systems was also comparable to that for hemin alone although with Amplex Red, hemin showed slightly higher activity. In contrast to ABTS reaction, fluorescence indicators were less effectively oxidized in the presence of Ref-hemin conjugate. However, Ref2-hemin exhibited the lowest activity compared with other systems. 

### 3.2. G4-Hemin Conjugates for Bioassay Development

**Scheme 1:**
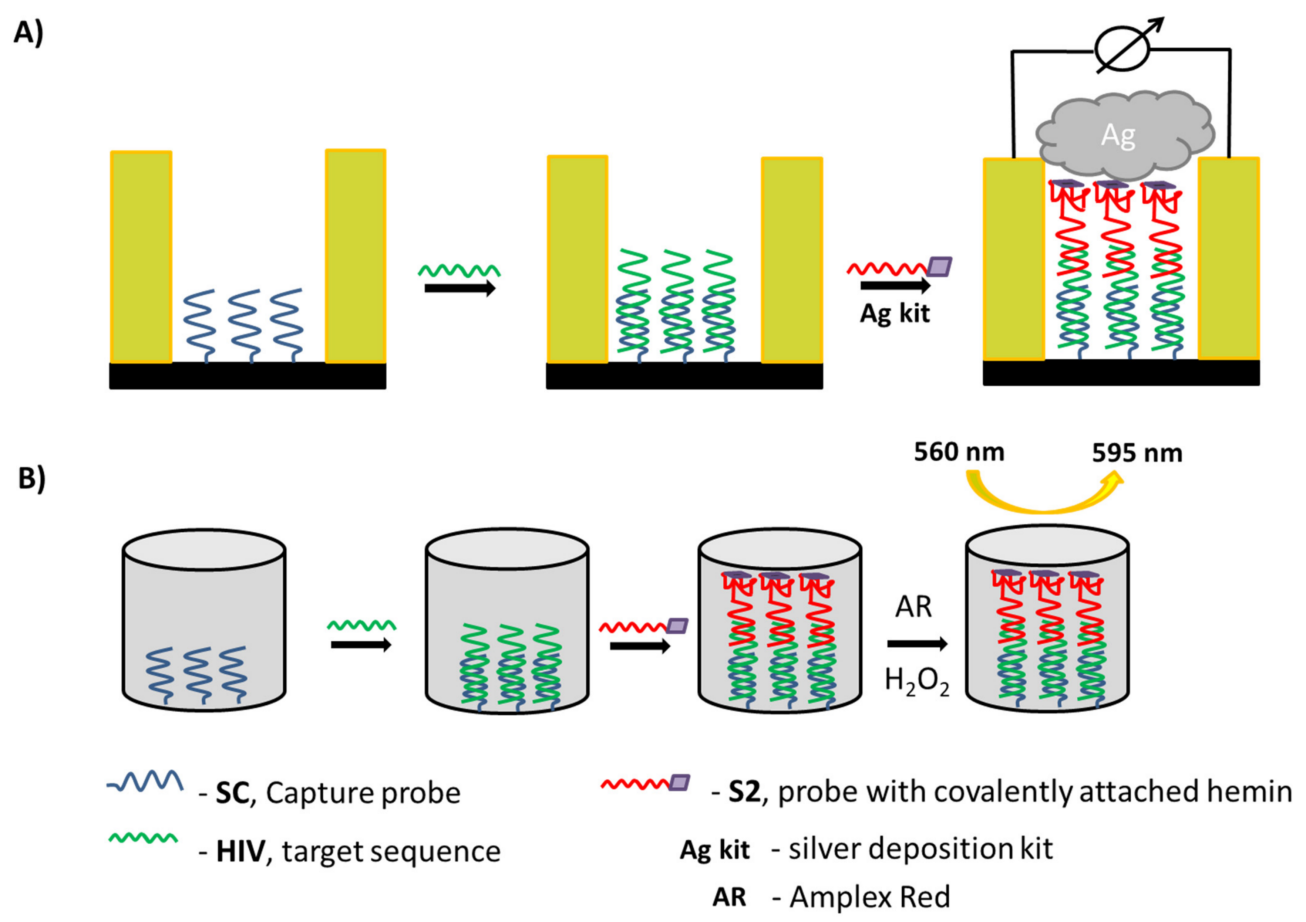
Scheme of HIV DNA sequence detection using electric chip, hybridization with a “sandwich” approach, and silver deposition catalyzed by the covalent DNAzyme (**A**) and scheme of HIV DNA detection using “sandwich” covalent DNAzyme assay using streptavidin-modified microplates (**B**).

Although many papers were published on the covalent attachment of hemin to DNA, to our knowledge no application study was undertaken yet. We decided to use G4-hemin conjugates as signal generator in the construction of bioassay for DNA sequence related to virus HIV (target DNA). For this purpose, we designed a “sandwich” DNA assay with electric readout. Electric chips were previously developed by Schuler et al. and used in the system with horseradish peroxidase [18]. The chip was constructed in such a way that between micro-constructed gold electrodes there were gaps that prevented flow of the electric current (Figure S2). On the glass surface of the chip an amino-modified oligonucleotide probe SC was immobilized between electrodes (Scheme 1A). This probe was complementary to the part of the DNA analyte. The second probe S2 possessed two domains: one complementary to the other part of the target DNA and a second one that could form G-quadruplex and had covalently attached hemin. Based on our activity results we have chosen a HT22 sequence that exhibits the highest activity among all studied covalent DNAzyme sequences. In the final step, the sandwiched DNAzyme could catalyze silver deposition and the deposited silver connected electrodes, which allowed the flow of the current. 

**Figure 4:**
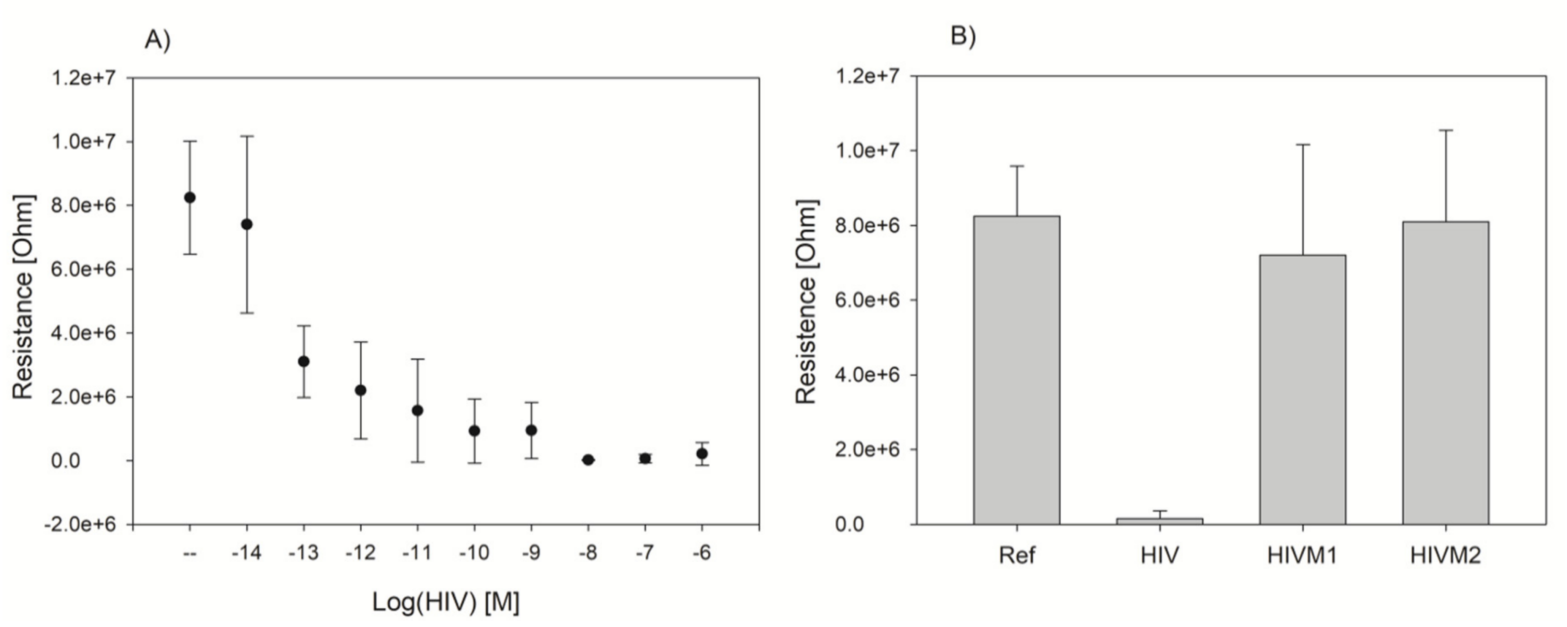
Concentration dependence of resistance measured on the electric chip for HIV DNA target sequence (**A**), selectivity of the method demonstrated on target DNA sequences with one (HIVM1) or two mismatches (HIVM2); samples were at 1 µM concentration (**B**).

We performed experiments with the electric chip and the “sandwich” DNAzyme assay using various concentrations of cDNA analyte in the range of 10^−6^–10^−14^ M. We observed that with the increasing concentration of DNA the resistance measured on the electric chip decreased (Figure 4). However, we were not able to quantify target, as we could not obtain a credible calibration plot covering the whole range of concentration tested. This was due to the variation of resistance for the blank probe and for samples with very low concentration of DNA. We have made an attempt to calculate limit of detection (LOD). Due to the negative slope, it was not possible to use directly a standard formula for LOD = 3.3Sd/S (Sd-standard deviation of blank probe, S-slope of the calibration curve). To calculate LOD we used method based on the graph approach. We calculated Sd for blank probe, multiplied it by 3.3 and then subtracted the value from the mean value of blank probe and finally we read concentration that corresponded to the obtained value of the signal. Based on this method we estimated the LOD to be 2 × 10^−13^ M. However, due to high standard deviations of measurement this method can be used for qualitative and not quantitative measurements. Therefore, we performed experiments with oligonucleotides containing one (HIVM1) and two mismatches (HIVM2) and we observed very good selectivity of the assay (Figure 4B). Based on these results, we conclude that the proposed method is rather sensitive for the detection of the analyte point mutation instead of DNA quantification. 

**Figure 5:**
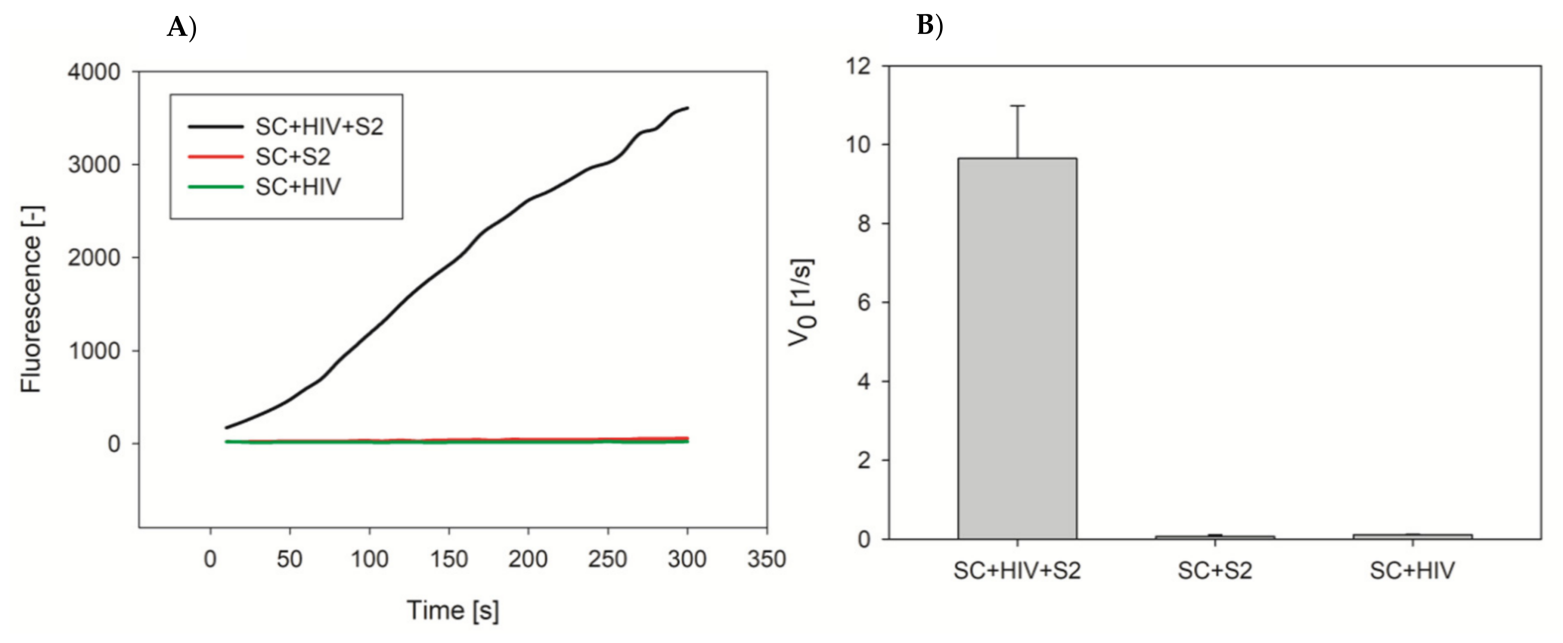
Kinetic plots for resorufin fluorescence generated from the Amplex Red oxidation reaction catalyzed by DNAzyme with covalently attached hemin molecule in a “sandwich” assay for detection of HIV DNA sequence (**A**) and initial velocity for the reaction with HIV DNA and negative probes (**B**). Conditions: 5 µM SC, 50 nM HIV DNA, 1 µM S2, 100 mM KCl, 10 mM Tris-HCl, 0.05% Tween 20, 1 µM Amplex Red, 1 mM H_2_O_2_.

The developed HIV DNA assay with the use of electric chip platform allowed limited application of the method for trace detection and quantification of target DNA. We decided then to perform preliminary experiments using fluorescence transduction approach in order to develop more accurate and sensitive DNA assay. For this purpose we used streptavidin-modified microplates and the same “sandwich” DNAzyme system (Scheme 1B). In this approach we used a biotinylated capture probe that was immobilized on the surface of well by streptavidin-biotin interaction. In the presence of target DNA, the capture probe hybridized with the part of the target oligonucleotide and the other part hybridized with the probe S2 possessing a domain with covalently attached hemin that formed DNAzyme. In the last step, the hybridized DNAzyme catalyzed peroxidation of Amplex Red to fluorescent resorufin. We compared the results of resorufin fluorescence measurement for the system with SC probe, HIV target sequence and S2 probe (positive control), with samples without HIV analyte (negative control), or without S2 probe (negative control). We obtained high signal difference between positive and negative controls (Figure 5). Based on these preliminary results we believe that this approach is more suitable for the development of biosensing system for DNA quantification at low concentration range. This were only preliminary results to prove potential of “sandwich” DNAzyme assay using probes with covalently attached hemin. The experiments will be further continued to evaluate sensitivity, dynamic range, and detection limit of the method. 

## 4. Discussion

In this study, we have examined the effect of DNA sequence on topology, thermal stability, pKa of hemin, and catalytic activity of DNA-hemin conjugates synthesized using click chemistry. Based on the CD experiments we can conclude that covalent attachment of hemin in most cases caused transformation of the original G4 topology into the parallel G-quadruplex. This is a crucial feature, since DNAzymes formed by parallel G-quadruplexes are known to possess higher activity than other structures [19]. Additionally, conjugation of hemin to DNA increased thermal stability of G-quadruplexes and for all studied conjugates T_m_ was higher than 80 °C. Based on the activity results we can conclude that the covalent conjugation of hemin was the most beneficial for oligonucleotide sequences that normally formed association DNAzyme complexes with low activity. The results are also in good agreement with determined pKa values since the decreases in pKa and activity were observed for PS2.M-hemin and CatG4-hemin and, respectively, increases were observed for HT22-hemin and TBA-hemin DNAzymes. Activity experiments were also performed using MNBDH as a substrate (Figure S3). The selectivity towards different substrates varied between DNAzymes based on different sequences but we also observed the highest increase of activity for conjugate HT22-hemin. 

It was reported by Wu et al. that single-stranded DNA can inhibit peroxidase activity of hemin in reaction with TMB [29,30]. According to their reports, DNA oligonucleotide may decrease catalytic activity of hemin by shielding the ferric ion as result of binding of pyrimidine and purine rings of nucleotides with hemin pyrrol rings. We did not observe such an effect with our oligonucleotide sequences for Ref/hemin and Ref2/hemin systems, but this effect was observed for Ref2-hemin when hemin was covalently attached to DNA. Our hypothesis is that covalent attachment of hemin to oligonucleotide restrict its liability, so the effect of potential inhibition is greater. In the case of Ref-hemin conjugate there can be two opposing effects: inhibition of hemin by single-stranded DNA and increase of activity due to guanine residues that can take part in electron transfer. Nakayama et el. previously studied DNA-hemin conjugates obtained by amino-coupling [14].They also observed some increase of peroxidase activity for oligonucleotide with random sequence. The proposed explanation involved binding of hemin to DNA oligonucleotide that helped with disaggregation of hemin and caused slightly higher activity of reference system. However, our results prove that the sequence of single-stranded DNA can have effect on peroxidase activity and guanine residues have important role in the increasing of activity of single stranded DNA-hemin conjugates. It should be also noted that observed effects depend on the used indicator.

In the second part of the manuscript, we designed the “sandwich” system for detection of DNA. For this purpose, we used electric chips and silver deposition reaction catalyzed by DNAzyme. Although the method developed by us could not be used for quantitative analysis the response was selective to oligonucleotides HIVM1 and HIVM2 that had mismatches. This method can be then used for qualitative analysis of genetic mutations. Electric chips have a very good potential to be adapted for use in the field since they do not require sophisticated instrumentation. In such environment methods for detection, for example, of pathogens, the generated positive or negative readouts are sufficient enough. We have chosen HIV DNA as a model analyte for proof-of-principle study, but this method can be easily extended by simple adaptation to detection of other DNA sequences. 

## 5. Conclusions

In this paper we studied the effect of the DNA sequence on topology, thermal stability, pKa, and peroxidase activity of DNA-hemin conjugates. Our results showed that conjugation of hemin in most cases promotes parallel the topology of G-quadruplex. Additionally, hemin conjugation strongly affects the thermal stability of G-quadruplexes and all studied G4-hemin conjugates in the presence of potassium had melting temperature above 80 °C. We noticed that pKa of hemin for oligonucleotides that forms G4/hemin association DNAzymes with high activity, after conjugation of hemin decreased, whereas pKa for oligonucleotides that normally formed association DNAzymes with low activity increased significantly after conjugation. As a consequence, the activity for DNAzymes containing HT22 and TBA sequences, which possessed very low activity in the association approach exhibited high peroxidase activity, after conjugation of hemin. These results proved that pKa is the factor that correlates well with the activity of DNAzymes. 

We have developed a bioassay for DNA detection using DNAzymes with covalently attached hemin. Based on our knowledge, this is the first attempt to use covalent DNAzymes for analytical applications. First, we designed the sensing system for DNA detection using electric chip. The results proved that the method is selective and can be used for detection of DNA single nucleotide mutation, which can be useful in point-of-care (POC) diagnosis. To further prove the effectiveness of our “sandwich” DNAzyme platform, we performed preliminary experiments using streptavidin-modified microplates and a fluorescence detection approach. These results showed that the system worked very well using microplates and that the approach can be exploited for the development of sensitive and selective bioassay for DNA detection with a low detection limit. 

## Figures and Tables

**Table 1:** Sequences of oligonucleotides used in this study.

Name	Sequence	Function
PS2.M	5′ GTG GGT AGG GCG GGT TGG 3′	Hemin aptamer [7]
PS2.M-hemin	hemin-5′ GTG GGT AGG GCG GGT TGG 3′	DNAzyme
CatG4	5′ TGG GTA GGG CGG GTT GGG AAA 3′	Hemin aptamer [19]
CatG4-hemin	hemin-5′ TGG GTA GGG CGG GTT GGG AAA 3′	DNAzyme
AGRO100	5′ GGT GG TGG TGG TTG TGG TGGTGGTGG 3′	Nucleolin aptamer [20]
AGRO100-hemin	hemin-5′ GGT GG TGG TGG TTG TGG TGG TGG TGG 3′	DNAzyme
TBA	5′ GGT TGG TGT GGT TGG 3′	Thrombin aptamer [21]
TBA-hemin	hemin-5′ GGT TGG TGT GGT TGG 3′	DNAzyme
HT22	5′ A GGG TTA GGG TTA GGG TTA GGG 3′	Human telomeric sequence [22]
HT22-hemin	hemin-5′ A GGG TTA GGG TTA GGG TTA GGG 3′	DNAzyme
Ref	5′ GAG TAG TTC GTG GCC TAG 3′	Reference sequence
Ref-hemin	hemin-5′ GAG TAG TTC GTG GCC TAG 3′	Reference conjugate
Ref2	5′ TTT ATT TAT TTA TTT ATT TAT T 3′	Reference sequence
Ref2-hemin	hemin-5′ TTT ATT TAT TTA TTT ATT TAT T 3′	Reference conjugate
SC	5′-GTG TGG AAA ATC TCT AGC AGT TTT-3′-NH_2_/biotin	Capture probe
S2	hemin-5′-AGG GTT AGG GTT AGG GTT AGG GTC CCT CAG ACC CTT TTA GTC-3′	Detection probe
HIV	5′-ACT GCT AGA GAT TTT CCA CAC TGA CTA AAG GGT CTG AGG GA-3′	Analyte, sequence of virus HIV
HIVM1	5′-ACT GCT AGA GAT TCT CCA CAC TGA CTA AAG GGT CTG AGG GA-3′	Analyte with 1 mismatch
HIVM2	5′-ACT GCT GGA GAT TCT CCA CAC TGA CTA AAG GGT CT GAG GGA-3′	Analyte with 2 mismatches

**Table 2:** Melting temperatures of studied oligonucleotides in the presence of potassium or sodium cations measured using CD technique ^1^.

	G4		G4/Hemin		G4-Hemin	
	Na^+^	K^+^	Na^+^	K^+^	Na^+^	K^+^
PS2.M	46.0	56.5	45.2	64.6	58.4	>80
CatG4	41.8	79.2	42.2	>80	53.0	>80
AGRO100	35.7	64.9	36.9	65.7	38.6	>80
HT22	53.9	62.7	52.9	63.4	80.0	>80
TBA	23.3	50.7	21.5	49.0	57.8	>80

^1^ Error for all of melting temperature is ±0.5 °C.

**Table 3:** The pKa values determined for DNA/hemin complexes and DNA-hemin conjugates.

Name	pKa	
	G4/Hemin	G4-Hemin
PS2.M	>10 ^1^	8.71
CatG4	>10 ^1^	8.78
AGRO100	9.6	9.73
HT22	7.91	>10 ^1^
TBA	8.9	>10 ^1^

^1^—for the measurements where pKa was too high to determine exact value we estimated pKa > 10.

## Data Availability

All data are provided in the manuscript and Supplementary Materials.

## Supplementary Materials

The following are available online at https://www.mdpi.com/article/10.3390/s22020500/s1, Figure S1: Figure S1. CD spectra of DNA oligonucleotide (black), DNA/hemin complexes, DNA-hemin conjugates in the presence of sodium cations for various DNA sequences: PS2.M (A), CatG4 (B), AGRO100 (C), HT22 (D) and TBA (E). Conditions: 2 µM DNA, 100 mM KCl, 10 mm Tris-HCl (pH = 8.0), Figure S2: Layout of the electric chip developed and produced in Institute of Photonic Technology, Figure S3: Initial velocity of ABTS oxidation by hemin, DNA/hemin complexes and DNA-hemin conjugates in various cationic conditions: 100 mM KCl (black), 100 mM NaCl (red), without cations (green).
