# Supplementary material for: Sequence Effect on the Activity of DNAzyme with Covalently Attached Hemin and Their Potential Bioanalytical Application
Source: Sensors (Basel). 2022 Jan 10;22(2):500. doi: 10.3390/s22020500 (PMC8780643; doi:10.3390/s22020500)
Supplement: Supplementary file 1 [file sensors-22-00500-s001.zip › sensors-1510927-supplementary.pdf]

Electronic supplementary information

Effect of sequence on activity of DNAzyme with covalently attached hemin and their potential bioanalytical application

J. Kosman[1*], K. Żukowski[1], A. Csaki[2], W. Fritzsche[2], B. Juskowiak[1]

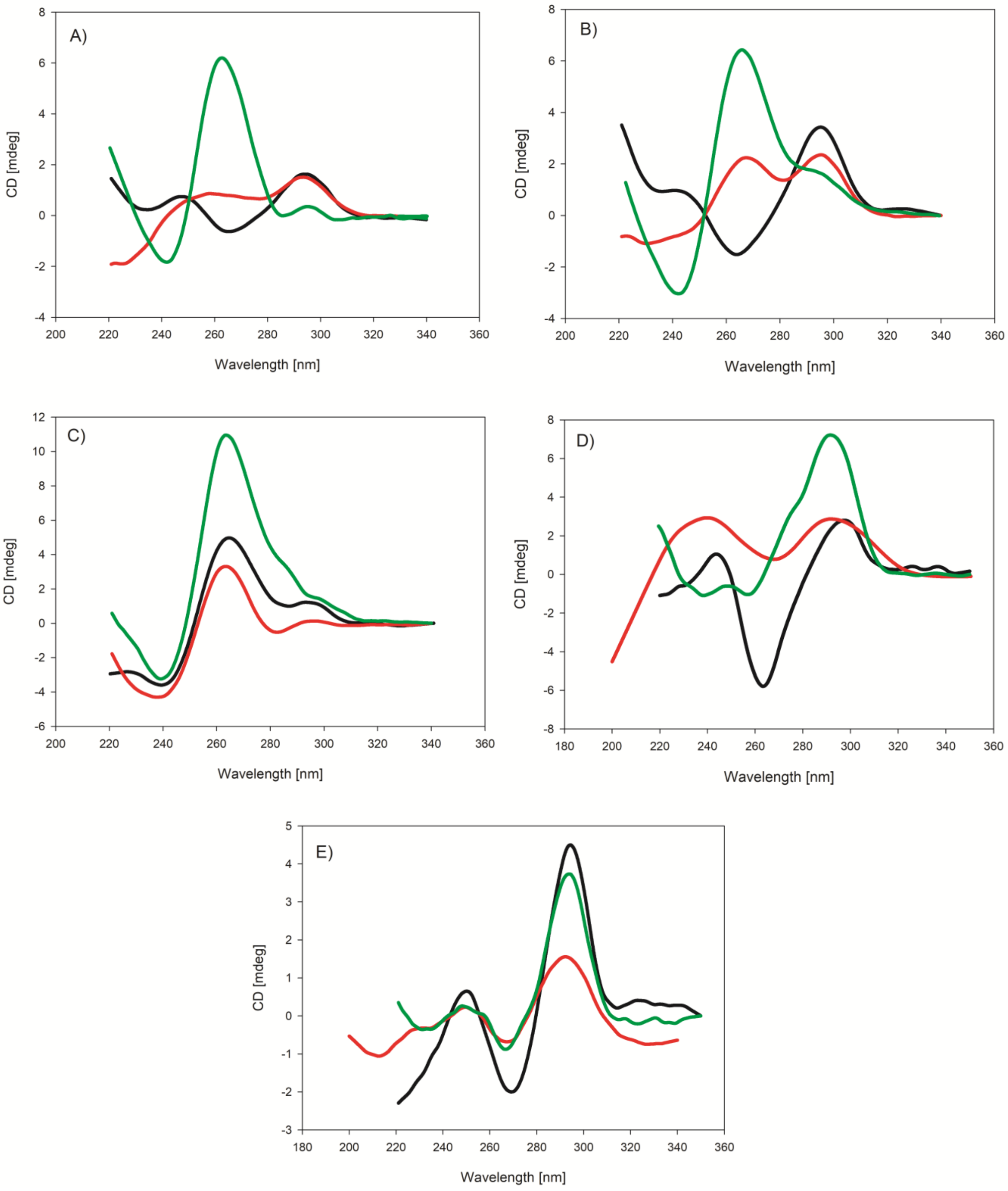

Figure S1. CD spectra of DNA oligonucleotide (black), DNA/hemin complexes, DNA-hemin conjugates in the presence of sodium cations for various DNA sequences: PS2.M (A), CatG4 (B), AGRO100 (C), HT22 (D) and TBA (E). Conditions: 2 μM DNA, 100 mM KCl, 10 mm Tris-HCl (pH=8.0).

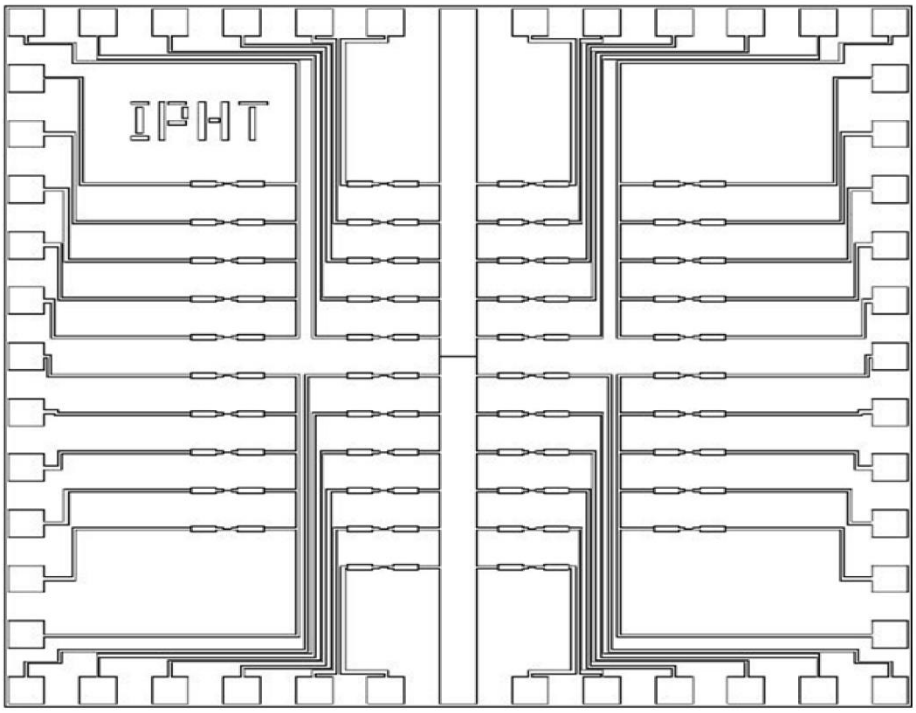

Figure S2. Layout of the electric chip developed and produced in Institute of Photonic Technology.

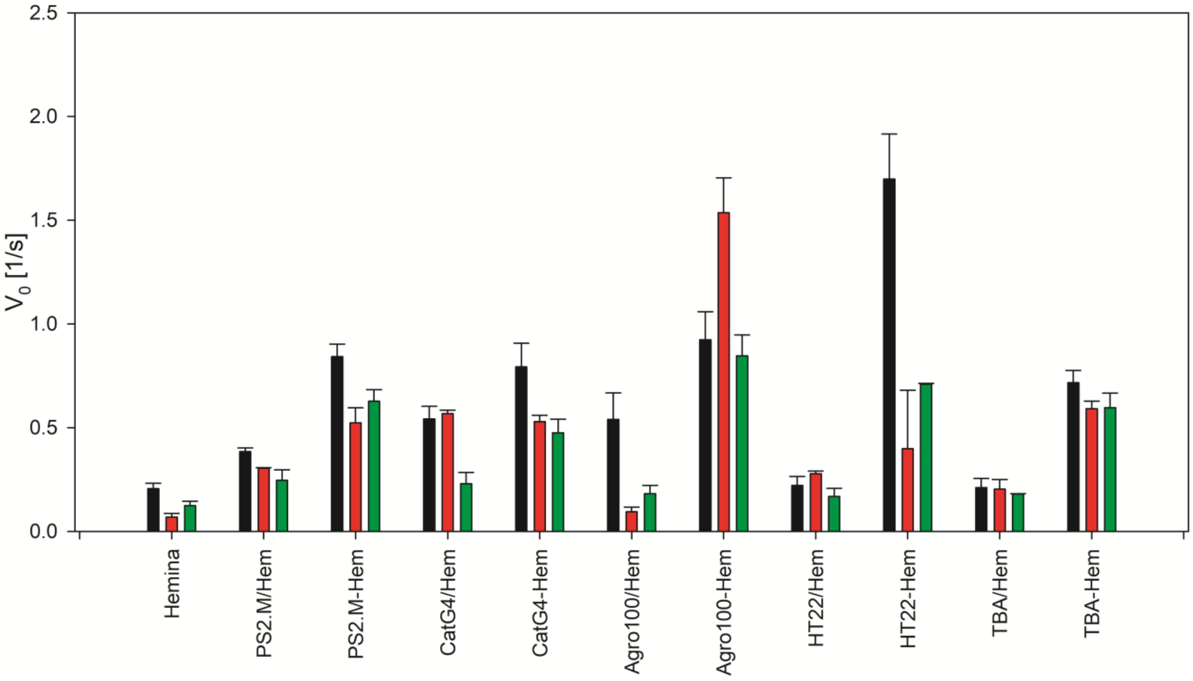

Figures S3. Initial velocity of ABTS oxidation by hemin, DNA/hemin complexes and DNA-hemin conjugates in various cationic conditions: 100 mM KCl (black), 100 mM NaCl (red), without cations (green).
